# *KIF15* missense variant is associated with the early onset of idiopathic pulmonary fibrosis

**DOI:** 10.1186/s12931-023-02540-0

**Published:** 2023-09-30

**Authors:** Maria Hollmén, Atte Laaka, Juulia J. Partanen, Jukka Koskela, Eva Sutinen, Riitta Kaarteenaho, Mari Ainola, Marjukka Myllärniemi

**Affiliations:** 1 Individrug, Heart and Lung Centre, The University of Helsinki and Helsinki University Hospital, Research Programs Unit, Helsinki, Finland; 2 Institute for Molecular Medicine Finland (FIMM), Helsinki Institute of Life Science (HiLIFE), University of Helsinki, Helsinki, Finland; 3 Research Unit of Biomedicine and Internal Medicine, University of Oulu and Oulu University Hospital, Oulu, Finland

**Keywords:** Idiopathic pulmonary fibrosis, *KIF15*, *TERT*, *SPDL1*, Missense variant

## Abstract

**Background:**

Idiopathic pulmonary fibrosis (IPF) has an unknown aetiology and limited treatment options. A recent meta-analysis identified three novel causal variants in the *TERT*, *SPDL1*, and *KIF15* genes. This observational study aimed to investigate whether the aforementioned variants cause clinical phenotypes in a well-characterised IPF cohort.

**Methods:**

The study consisted of 138 patients with IPF who were diagnosed and treated at the Helsinki University Hospital and genotyped in the FinnGen FinnIPF study.

Data on > 25 clinical parameters were collected by two pulmonologists who were blinded to the genetic data for patients with *TERT* loss of function and missense variants, *SPDL1* and *KIF15* missense variants, and a *MUC5B* variant commonly present in patients with IPF, or no variants were separately analysed.

**Results:**

The *KIF15* missense variant is associated with the early onset of the disease, leading to progression to early-age transplantation or death. In patients with the *KIF15* variant, the median age at diagnosis was 54.0 years (36.5–69.5 years) compared with 72.0 years (65.8–75.3 years) in the other patients *(P* = 0.023). The proportion of *KIF15* variant carriers was 9- or 3.6-fold higher in patients aged < 55 or 65 years, respectively. The variants for *TERT* and *MUC5B* had similar effects on the patient’s clinical course, as previously described. No distinct phenotypes were observed in patients with the *SPDL1* variant.

**Conclusions:**

Our study indicated the potential of *KIF15* to be used in the genetic diagnostics of IPF. Further studies are needed to elucidate the biological mechanisms of *KIF15* in IPF.

**Supplementary Information:**

The online version contains supplementary material available at 10.1186/s12931-023-02540-0.

## Background

Idiopathic pulmonary fibrosis (IPF) is a progressive lung disease that is accompanied by respiratory symptoms, primarily dyspnoea, and a poor quality of life. The impacts of current antifibrotic therapies are limited, and the prognosis has not improved sufficiently [1, 2].

Studies are slowly unravelling the aetiology of IPF with evidence of genetic susceptibility combined with external risk factors and possible exposure. More than 25 different genetic regions and numerous variants have been reported to be involved in IPF [3–7]. Although data on genetic variants are abundant, little is known about their clinical significance. The clinical aspects have been best described in variants in the telomerase and promoter regions of *MUC5B*. A common variant in *MUC5B* has the largest effect on IPF risk, but rare variants, such as *TERT*, are considered more important in disease pathogenicity [5, 7, 8]. Variants in non-telomerase-related genes, such as *SDPL1*, *KIF15,* and surfactant-related genes, have recently been suggested to be of importance in IPF [5, 7, 9–11]. These single studies focused on genetic data with limited clinical features. There is a lack of studies where detailed clinical data are presented along with the genetic variants.

A recent large-scale meta-analysis of IPF genetics suggested causal coding variants at three loci –*TERT*, *SPDL1*, and *KIF15,* in the FinnGen IPF (FinnIPF) population [12]. The unique genetic background of an isolated population in Finland has provided a special basis for several genetic research studies [13, 14]. Using data from the national biobank and large GWAS studies, we aimed to correlate this genetic data with a well-defined clinical cohort of 138 patients with IPF.

We studied the effects of the predicted rare causal variants of *TERT*, *SPDL1*, and *KIF15* and the common variant of *MUC5B* on the clinical phenotype and disease course of patients with IPF.

## Methods

The FinnishIPF study is a nationwide epidemiological registry study [15]. The inclusion criteria are written informed consent and a diagnosis of IPF according to the ATS/ERS criteria [16]. FinnGen (https://www.finngen.fi/en) is a large biobank study in which diagnoses are based on ICD codes. FinnGen samples were genotyped using multiple Illumina and Affymetrix arrays (Illumina, San Diego, CA, USA and Thermo Fisher Scientific, Santa Clara, CA, USA, respectively), filtered, and imputed with a population-specific reference panel as previously described [15].

In 2017, a subgroup of patients in the FinnishIPF study was contacted to collect DNA and serum samples and was included in the FinnGen analysis (FinnIPF cohort, *N* = 235). Our study cohort consisted of FinnishIPF study patients who were followed up at the Helsinki University Hospital (*N* = 138).

In the present study, causal variants predicted by fine-mapping in the FinnGen study, two *TERT* loss of function (Lof) and missense variants (rs770066110 and rs776981958) and *SPDL1* (rs116483731) and *KIF15* missense (rs138043992) variants, were included in the analyses [12]. We also analysed the most well-known genetic variant for IPF, *MUC5B* (rs35705950) [4, 9, 17]. Due to the extremely high prevalence (97/138), the patients with the *MUC5B* variant were investigated separately.

Using electronic medical records and CT scans at the Helsinki University Hospital, we collected pre-specified clinical characteristics of our patient cohort (*N* = 138) in July 2022. Two pulmonologists specialising in interstitial lung diseases re-evaluated these clinical characteristics. The genetic data were blinded to the pulmonologists and added to the analysis after the clinical evaluation was completed by unblinded researchers. Before the unblinding and analysis, three patients (2.1%, 3/138) were excluded from the final cohort: one did not have IPF, and two were immigrants with a genetically remote ethnicity. None of the excluded patients possessed any of the genetic variants under investigation.

### Statistical analysis

All patients with either *KIF15*, *TERT*, or *SPDL1* missense variants were grouped according to variant status, and the patients without any of the studied variants were grouped as “no Qv”. One of the patients (1/135) harboured two of the studied variants (*TERT* and *SPDL1* missense) and was excluded from the analyses that simultaneously included both the *TERT* and *SPDL1* missense groups to maintain the independence of the groups necessary for statistical testing.

We used a Kruskal–Wallis or Fisher's exact test (with a Freeman–Halton extension for contingency tables larger than 2 × 2) to evaluate the differences between variant groups, depending on the variable type. Furthermore, the variant groups were individually compared with the no Qv group using a Mann–Whitney U or Fisher’s exact test. As the differences between the *KIF15* and no Qv groups were consistent, the *KIF15* group was further compared with a group consisting of all other patients. We also conducted Kaplan–Meier one-minus survival and survival analyses using the Mantel–Cox log-rank test to estimate disease occurrence and progression. In these analyses, the exact timing and occurrence of events (diagnosis, death, or transplantation) were known without any missing data or dropouts. As a result, only the patients who survived to the end of the study period without death or transplantation were censored (no patients were censored in the disease occurrence analysis, as all patients were diagnosed with IPF).

All data are presented as median (IQR) or % (*n*/*N*), and a two-tailed *P* < 0.05 was considered significant. Statistical analyses were performed using IBM SPSS Statistics (version 25, SPSS Inc., Chicago, IL, USA).

## Results

The patient characteristics of the variant groups are shown in Table 1. Most patients were male and smokers with no family history of pulmonary fibrosis. Comorbidities included hypertension, hyperlipidaemia, coronary artery disease, obesity, type 2 diabetes mellitus, and hypothyroidism, and 33 patients had cancer: 12 lung, 11 prostate, 6 gastrointestinal tract, 4 bladder, 3 breast, 1 cervix, and 1 renal cancer. The variant groups differed, especially in variables relating to treatment and prognosis, such as the probability of lung transplantation, age at diagnosis, or death (Table 1).

**Table 1:** Patient characteristics of all patients and by variant groups, and the statistical comparison between variant groups, KIF15 missense, SPDL1 missense, TERT, and No Qv (patients without any of these variants)

	All patients (*N* = 135)	*KIF15* missense (*N* = 5)	*SPDL1* missense (*N* = 21)	*TERT* (*N* = 8)	No Qv (*N* = 100)	Sig
Background and comorbidities						
Female sex	43 (32%)	0 (0%)	7 (33%)	1 (13%)	35 (35%)	0.313
Male sex	92 (68%)	5 (100%)	14 (67%)	7 (88%)	65 (65%)	0.313
Family history of pulmonary fibrosis	8 (6%)	0 (0%)	1 (5%)	2 (25%)	5 (5%)	0.173
Smoking history	86 (64%)	4 (80%)	17 (81%)	5 (63%)	59 (59%)	0.250
Cancers	33 (24%)	1 (20%)	6 (29%)	1 (13%)	25 (25%)	0.899
BMI, kg/m^2^	28.0 (25.0–30.0)	34.0 (28.1–36.0)	28.0 (25.4–31.1)	27.4 (25.2–32.8)	27.3 (24.9–30.0)	0.131
Comorbidities, N	4.0 (2.0–6.0)	5.0 (2.0–7.5)	3.0 (1.5–7.0)	3.5 (0.5–4.8)	4.0 (2.0–6.0)	0.450
Treatment and prognosis						
Antifibrotic treatment	79 (59%)	4 (80%)	10 (48%)	4 (50%)	60 (60%)	0.519
Lung transplantation	15 (11%)	2 (40%)	1 (5%)	3 (38%)	8 (8%)	0.013*
Oxygen therapy	43 (32%)	4 (80%)	7 (33%)	3 (38%)	29 (29%)	0.126
Acute exacerbations	34 (25%)	1 (20%)	5 (24%)	1 (13%)	27 (27%)	0.925
Deceased	62 (46%)	1 (20%)	12 (57%)	2 (25%)	46 (46%)	0.318
Age at diagnosis, years	71.0 (65.0–75.0)	54.0 (36.5–69.5)	68.0 (65.0–76.0)	67.0 (59.3–70.3)	72.0 (66.0–76.0)	0.016*
Age at death, years	78.8 (74.1–83.0)	61.2 (61.2–61.2)	77.5 (69.9–80.2)	74.1 (71.3–74.1)	79.6 (74.7–84.2)	0.075
Age at transplant, years	61.1 (49.8–65.0)	40.2 (30.9–40.2)	63.5 (63.5–63.5)	68.0 (63.7–68.0)	57.4 (50.7–61.6)	0.033*
Age at death or transplant, years	76.5 (68.5–81.4)	49.4 (30.9–49.4)	76.3 (68.7–80.0)	68.7 (64.8–74.9)	78.6 (72.3–83.8)	0.013*
Symptoms at diagnosis						
Dyspnoea	68 (50%)	3 (60%)	13 (62%)	3 (38%)	49 (49%)	0.637
Cough	69 (51%)	1 (20%)	8 (38%)	4 (50%)	55 (55%)	0.269
Laboratory findings at diagnosis						
Macrocytosis	30 (22%)	1 (20%)	5 (24%)	2 (25%)	21 (21%)	0.942
Thrombocytopenia	18 (13%)	1 (20%)	2 (10%)	1 (13%)	13 (13%)	0.895
Radiological findings at diagnosis						
Traction bronchiectasis	132 (98%)	5 (100%)	21 (100%)	8 (100%)	97 (97%)	1.000
Honeycombing	108 (80%)	4 (80%)	15 (71%)	4 (50%)	85 (85%)	0.050*
Ground-glass opacity	25 (19%)	2 (40%)	4 (19%)	0 (0%)	18 (18%)	0.324
Right ventricular strain	17 (13%)	1 (20%)	3 (14%)	0 (0%)	13 (13%)	0.697
Emphysema	32 (24%)	1 (20%)	6 (29%)	1 (13%)	24 (24%)	0.896
Pulmonary function at diagnosis						
FVC, L	2.96 (2.42–3.70)	3.35 (2.77–3.58)	2.97 (2.52–3.48)	3.41 (2.71–4.22)	2.90 (2.29–3.76)	0.468
FVC, % predicted	81.0 (68.0–93.0)	59.0 (51.5–90.0)	79.0 (69.5–89.5)	73.0 (64.3–97.5)	84.5 (70.3–94.8)	0.327
FEV1, L	2.37 (2.00–3.00)	2.73 (2.27–2.76)	2.21 (2.08–2.72)	2.55 (2.20–3.57)	2.34 (1.91–3.04)	0.587
FEV1, % predicted	80.0 (72.8–94.3)	66.0 (55.0–84.0)	77.0 (72.5–90.5)	77.0 (62.0–103.0)	83.0 (74.0–95.8)	0.275
DLCO, % predicted	59.0 (50.0–69.3)	52.0 (37.0–64.5)	56.0 (45.5–70.5)	62.5 (47.5–75.5)	60.0 (52.0–69.0)	0.423

Bold values are statistically significant *p* < 0.05

### *KIF15* missense variant

Patients with the *KIF15* missense variant were the youngest at the time of diagnosis, transplantation, death, or both endpoints combined (Table 1).

They were diagnosed at an earlier age (median 54.0 years, 36.5–69.5, *n*_*1*_ = 5) compared with those without any of the studied variants (72.0 years, 66.0–76.0, *n*_*2*_ = 100, *U* = 99.5, *P* = 0.023). They also died at a younger age (61.2 years, *n*_*1*_ = 1) than the patients in the no Qv group (72.0 years, 66.0–76.0, *n*_*2*_ = 100, *U* = 99.5*, P* = 0.023). When both endpoints (transplantation and death) were combined, the patients with the *KIF15* missense variant were noticeably younger (49.4 years, 30.9–49.4, *n*_*1*_ = 3) compared with those in the no Qv group (78.6 years, 72.3–83.8, *n*_*2*_ = 53, U = 7.0*, P* = 0.002).

**Fig. 1:**
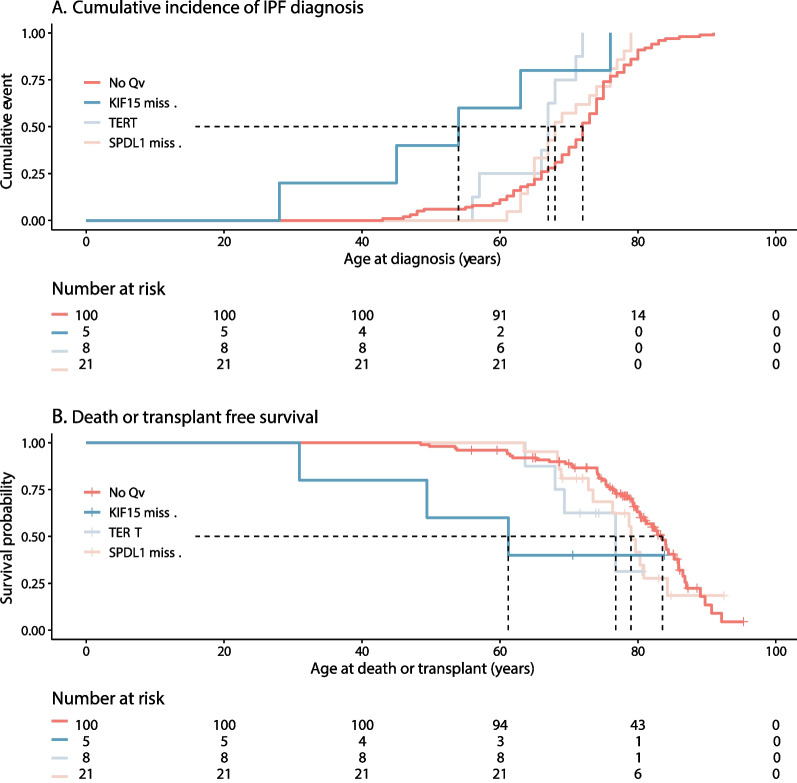
**A**, **B** Disease progression in the *KIF15* missense, *TERT*, *SPDL1* missense, and No Qv groups ("No Qv", patients without studied *TERT*, *SPDL1*, and *KIF15* missense variants)

According to the Kaplan–Meier one-minus survival analysis, the IPF occurred significantly earlier among the patients with the *KIF15* missense variant compared with those in the no Qv group (χ^2^(1) = 6.53*, P* = 0.011, Fig. 1A). In addition, the death or transplant-free survival was significantly weaker in the *KIF15* missense group compared with that in the no Qv group (χ^2^(1) = 3.96*, P* = 0.047, Fig. 1B). The *KIF15* missense group also clearly stood out from others in terms of death and transplant-free survival (Fig. 1B).

Patients with the *KIF15* missense variant had a higher median body mass index (BMI) of 34.0 (28.1–36.0, *n*_*1*_ = 5) than those in the no Qv group (27.3, *n*_*2*_ = 100, U = 398.5*, P* = 0.025). Most patients with the *KIF15* missense variant, 80% (4/5), were started on supplemental oxygen therapy, relative to the 29% (29/100) of the no Qv patients (*P* = 0.033).

As the differences between the patients in the *KIF15* missense and no Qv groups were consistent, we compared the *KIF15* group to those without *KIF15* variant carriers (“Others”). The results were even more pronounced when patients with other variants were included in the analyses (Table 2). The patients in the *KIF15* group were diagnosed (*U* = 129.5*, P* = 0.023), died (*U* = 0.0*, P* = 0.032), and either received the transplant or died (*U* = 7.0*, P* = 0.001) at a significantly younger age than those of the others. In addition, the patients with the *KIF15* variant needed oxygen therapy more often than the other patients (*P* = 0.036).

**Table 2:** Patient characteristics and the statistical comparison between patients with the KIF15 missense variant and all other patients

	KIF15-missense (*N* = 5)	Others (*N* = 130)	Sig
Background and comorbidities			
Female sex	0 (0%)	43 (33%)	0.177
Male sex	5 (100%)	87 (67%)	0.177
Family history of pulmonary fibrosis	0 (0%)	8 (6%)	1.000
Smoking history	4 (80%)	82 (63%)	0.653
Cancers	1 (20%)	32 (25%)	1.000
BMI, kg/m^2^	34.0 (28.1–36.0)	27.8 (25.0–30.0)	0.028*
Comorbidities, N	5.0 (2.0–7.5)	4.0 (2.0–6.0)	0.432
Treatment and prognosis			
Oxygen therapy	4 (80%)	39 (30%)	0.036*
Antifibrotic treatment	4 (80%)	75 (58%)	0.402
Lung transplantation	2 (40%)	13 (10%)	0.095
Deceased	1 (20%)	61 (47%)	0.374
Age at diagnosis, years	54.0 (36.5–69.5)	72.0 (65.8–75.3)	0.023*
Age at death, years	61.2 (61.2–61.2)	79.0 (74.1–83.2)	0.032*
Age at transplant, years	40.2 (30.9–40.2)	61.8 (53.6–65.2)	0.038*
Age at death or transplant, years	49.4 (30.9–49.4)	76.8 (69.3–82.0)	< 0.001*
Symptoms			
Dyspnoea	3 (60%)	65 (50%)	1.000
Cough	1 (20%)	68 (52%)	0.202
Acute exacerbations	1 (20%)	33 (25%)	1.000
Laboratory findings			
Macrocytosis	1 (20%)	29 (23%)	1.000
Thrombocytopenia	1 (20%)	17 (13%)	0.520
Radiological findings			
Right ventricular strain	1 (20%)	16 (12%)	0.495
Emphysema	1 (20%)	31 (24%)	1.000
Honeycombing	4 (80%)	104 (80%)	1.000
Traction bronchiectasis	5 (100%)	127 (98%)	1.000
Ground-glass opacity	2 (40%)	23 (18%)	0.231
Pulmonary function test findings			
FVC, L	3.35 (2.77–3.58)	2.95 (2.40–3.71)	0.578
FVC, % predicted	59.0 (51.5–90.0)	81.0 (68.0–93.0)	0.128
FEV1, L	2.73 (2.27–2.76)	2.36 (1.96–3.04)	0.562
FEV1, % predicted	66.0 (55.0–84.0)	82.0 (73.0–95.0)	0.086
DLCO, % predicted	52.0 (37.0–64.5)	59.0 (50.5–69.5)	0.188

Bold values are statistically significant *p* < 0.05

Considering the timing of disease onset, the proportion of *KIF15* missense carriers was noticeably high among all the patients with early diagnosis: 50.0% (1/2) of patients with diagnosis before 45 years, 33.3% (3/9) of patients with diagnosis before 55 years, and 13.3% (4/30) of patients with diagnosis before 65 years. The total proportion of *KIF15* missense variant carriers in the study population was 3.7% (5/135).

### Other variants

The patients with the *SPDL1* variant died at a younger age (76.3 years, 68.7–80.0, *n*_*1*_ = 13) than those in the no Qv group (79.6 years, 74.7–84.2, *n*_*2*_ = 46, *U* = 412.0, *P* = 0.039).

Four and five patients possessed a *TERT* Lof and a *TERT* missense variant, respectively. These patients were pooled for statistical analysis. The proportion of patients reporting dyspnoea at the time of diagnosis (75.0% for Lof and 0.0% for missense) was the only parameter that differed among the groups (*P* = 0.048).

The patients with the *TERT* variants were younger (67.0 years, 58.5–69.5, *n*_*1*_ = 9) at the time of diagnosis than the no Qv patients (72.0 years, 66.0–76.0, *n*_*2*_ = 100, U = 678.5, *P* = 0.012). In addition, the cumulative incidence of IPF diagnosis differed from that among the no Qv patients (χ^2^(1) = 11.62, *P* = 0.001). Patients with *TERT* variants received a lung transplant more often (44.4%, 4/9) compared with the 8.0% of patients with no Qv (8/100, *P* = 0.008). They were also younger at the time of death or transplantation (68.0 years, 64.3–73.1, *N*_*1*_ = 5) compared with the no Qv patients (78.6 years, 72.3–83.8, *N*_*2*_ = 53, *U* = 61.0, *P* = 0.047). Patients with the *TERT* variants had lesser honeycombing in the HRCT (44.4%, 4/9) than no Qv patients (85.0%, 85/100, *P* = 0.010).

The *MUC5B* variant was found in 70% (97/138) of the patients. Due to its high prevalence in the total sample and the variant groups, it was not included in the variant group comparisons. Additional file 1: Table S1 shows the patient characteristics of the *MUC5B* variant group and other patients; the only significant difference was that the MUC5B carriers were significantly older at the time of transplantation than other patients. These groups did not differ regarding the prevalence of the other studied variants (p = 0.548 for *KIF15* missense, p = 0.537 for *SPDL1* missense, and p = 0.720 for *TERT* variants). The exclusion of patients with any of the other variants (*KIF15* missense, *SPDL1* missense, *TERT* variants) did not significantly change the results in any of the studied.

## Discussion

We investigated the possibility of defining a clinical IPF phenotype based on distinct genetic variants associated with IPF susceptibility. To the best of our knowledge, this is one of the first studies in which systematically collected clinical characteristics were compared with a detailed genetic evaluation.

This study showed that the *KIF15* missense variant (rs138043992) was associated with markedly early disease onset, a high need for supplemental oxygen, and disease progression to transplantation or death at an early age.

The studied *KIF15* missense variant causes a nucleotide substitution from arginine to leucine at the 501st amino acid (p.Arg501Leu). Hence, it could affect the stability and function of the KIF15 protein. KIF15 is a motor protein involved in mitotic spindle assembly and affects cell proliferation. It is upregulated in various cancers, including breast and gastric cancer. Overexpression of *KIF15* promotes lung carcinogenesis and cancer cell proliferation and is associated with a poor prognosis in non-small cell lung carcinoma [18–20]. Several *KIF15* variants (missense, Lof, and intron) have also been associated with IPF risk, but their impact on disease course and phenotype remains unclear [4, 10, 11].

The *KIF15* variant in this study was suggested to be causal in the fine-mapping of the FinnGen IPF population [12]. It was recently observed that variants in the Finnish population that were enriched by more than twofold were 1.7-fold more likely to be associated with a phenotype expected by chance [14]. Allelic frequency (AF) in our IPF cohort was 0.018, which showed a twofold increase compared with that of the reference Finnish population (AF 0.009), over fourfold compared with that of the non-Finnish Europeans (AF 0.004), and sixfold compared with that of ALL individuals (AF 0.003) based on the whole genome variant set version 3.1.2 of the Genome Aggregation Database (GnomAD). This shows that the *KIF15* missense variant allele is enriched in the Finnish population and could be a possible causal variant for IPF, as shown earlier [12].

Our main finding was that patients with the *KIF15* missense variant were diagnosed at a substantially younger age than other patients. The difference in the median diagnosis age between the *KIF15* variant carriers and others was large (over 10 years) and not explained by family history of IPF or statistical outliers. The proportion of *KIF15* variant carriers was ninefold higher in patients aged < 55 years. Our findings imply a potential link between the early onset of disease and the *KIF15* missense variant. Among the three earlier studies reporting on *KIF15* and *IPF*, only Zhang et al. reported the median age of the patients [4, 10, 11]. In their study, the median age of patients with *KIF15* variants (stop gain, missense, splice acceptor, and frameshift) was 64 years (*N* = 12), and that of all patients was 67 years. The study included both patients with IPF and other interstitial lung diseases [11]. It is important to note that the comprehensive *KIF15* mutation variant list in the Zhang data doesn’t include our *KIF15* variant found in an all-Finnish population.

Our *KIF15* missense variant patients received transplantation or died at a younger age than other patients. This was probably explained by the early onset of the disease and increased morbidity. Patients diagnosed at a younger age are more likely to meet the requirements for lung transplantation, and human lifespan is limited and influenced by many factors other than the variants (such as the presence and severity of comorbid conditions, smoking, and exposures). Hence, the large difference in the timing of disease onset has the potential to mask the effects of the variant on overall survival. Some results might suggest that the *KIF15* variant impacts the disease course. Patients with the *KIF15* variant needed oxygen therapy markedly more often than the others. Previously, Allen et al. reported an association between lower pulmonary function and the *KIF15* variant, rs78238620 [4]. In our study, the *KIF15* missense group performed the worst in pulmonary function tests, but probably due to the sample size, the results did not reach statistical significance. This might also obscure the finding that none of the patients with the *KIF15* missense variant had a known family history of pulmonary fibrosis despite the early onset of the disease. More studies on the possible effects of *KIF15* variants on disease course, mortality, and familial forms of the disease are needed.

Considering the notable effect on disease onset and the possible effect on disease progression, our findings support the view of Moss and Rosas, who highlighted that KIF15 has the potential to uncover disease mechanisms and even contribute to drug discovery [7]. Many centres employ genetic panels for clinical diagnosis, and the identified mutations influence clinical decisions [21, 22]. Our findings suggest that *KIF15* could be included in genetic screening panels.

The findings on the other, better-described variants (in *TERT*, *SPDL1,* and *MUC5B*) were in line with earlier studies. Telomerase reverse transcriptase (TERT) maintains telomere length by adding nucleotides to the ends of telomeres and plays a role in cellular senescence and cancer development. Shorter telomere lengths have been associated with IPF and poor survival [23–25]. The *TERT* variant was also associated with worse outcomes in our study. *SPDL1* variants are reportedly upregulated in the lung tissue of patients with IPF [9]. We did not find a distinct clinical phenotype for the *SPDL1* variant. Previously, Dhindsa et al. reported a summary of the clinical features of 26 IPF patients with the *SPLD1* variant without a clear or significant difference from other study patients. These results suggest that the *SPDL1* variants do not have any major impact on the clinical phenotype of IPF. The best-known single genetic risk factor for IPF is a variant in *MUC5B,* which is present in more than half of the patients with IPF and has been associated with susceptibility to IPF and a mild disease course [26–29]. In our study, 70% of the patients carried the *MUC5B* variant, and these patients needed lung transplantation at an older age than other patients. The other variants did not affect this result.

Our study has several strengths. Repeated genetic bottlenecks and isolation from other countries create a distinct genetic profile for the Finnish people, providing a unique setting for genetic studies. Patient samples were genotyped as part of a large FinnGen IPF study, and all patients had IPF and met the ATS/ERS criteria for the disease. We had access to detailed clinical histories of all patients, and all data were reviewed by two pulmonologists specialising in IPF care. The number of patients was limited, but the statistical assumptions were met, and there were no distinct outliers in any of the variant groups. The number of censored patients in the survival analyses was minimal.

Our study also has limitations. Our patient population was relatively small and from a single university hospital. Only four variants were analysed; therefore, the significance of other potentially significant variants remains unknown. The small number of patients in some variant groups limits the possibilities of adjusting the analyses with confounding factors, such as disease severity, sex, age, and comorbidities, that are known to influence IPF survival. It was not possible to analyse the association between genetic variants and pulmonary function over time. The observatory and exploratory nature of the study limit final conclusions regarding distinct phenotypes. Larger confirmatory studies within multi-ancestry genetic populations and multiple cohorts are needed to confirm these results and detect possible milder effects caused by other variants.

## Conclusions

Our study highlights the importance of connecting clinical characteristics to genetic data. We found that the *KIF15* missense variant is associated with a remarkably early onset of the disease, leading to transplantation and death at an early age. Our findings suggest the potential of KIF15 to be used in diagnostic procedures, patient monitoring, and screening, especially among young patients. We also confirmed the previously identified clinical phenotypes of *TERT* variants and that many patients with IPF are genetically susceptible to the disease. In the future, larger studies on KIF15 focusing on clinical aspects, especially disease course and survival, are needed to confirm its significance.

### Supplementary Information

**Additional file 1.** **Table S1.** Characteristics of the MUC5B positive patients.

## Data Availability

A large portion of the data can be found in a meta-analysis by Partanen et al. [12]. The data can be shared upon request by the corresponding author. Our data is from a small population with a rare disease and patients in distinct groups of causal variants (*KIF15* and *TERT)*. Hence, variants and patient demographics are identity attributes, and according to legislation on data protection, it is not possible to share de-identified patient data.

## Abbreviations

IPF: Idiopathic pulmonary fibrosis
KIF15: Kinesin family member 15
MUC5B: Mucin 5b
SPDL1: Spindle apparatus coiled-coil protein 1
TERT: Telomerase reverse transcriptase

## References

[1] Raghu G, Chen SY, Yeh WS, Maroni B, Li Q, Lee YC (2014). Idiopathic pulmonary fibrosis in US Medicare beneficiaries aged 65 years and older: incidence, prevalence, and survival, 2001–11. Lancet Respir Med.

[2] Lederer DJ, Martinez FJ (2018). Idiopathic pulmonary fibrosis. N Engl J Med.

[3] Kaur A, Mathai SK, Schwartz DA (2017). Genetics in idiopathic pulmonary fibrosis pathogenesis, prognosis, and treatment. Front Med (Lausanne).

[4] Allen RJ, Guillen-Guio B, Oldham JM, Ma SF, Dressen A, Paynton ML (2020). Genome-wide association study of susceptibility to idiopathic pulmonary fibrosis. Am J Respir Crit Care Med.

[5] Peljto AL, Blumhagen RZ, Walts AD, Cardwell J, Powers J, Corte TJ (2023). Idiopathic pulmonary fibrosis is associated with common genetic variants and limited rare variants. Am J Respir Crit Care Med.

[6] Chen M, Zhang Y, Adams T, Ji D, Jiang W, Wain LV (2022). Integrative analyses for the identification of idiopathic pulmonary fibrosis-associated genes and shared loci with other diseases. Thorax.

[7] Moss BJ, Rosas IO (2023). Defining the genetic landscape of idiopathic pulmonary fibrosis: role of common and rare variants. Am J Respir Crit Care Med.

[8] Newton CA, Oldham JM, Applegate C, Carmichael N, Powell K, Dilling D (2022). The role of genetic testing in pulmonary fibrosis: a perspective from the pulmonary fibrosis foundation genetic testing work group. Chest.

[9] Dhindsa RS, Mattsson J, Nag A, Wang Q, Wain LV, Allen R (2021). Identification of a missense variant in *SPDL1* associated with idiopathic pulmonary fibrosis. Commun Biol.

[10] Zhang D, Povysil G, Kobeissy PH, Li Q, Wang B, Amelotte M (2022). Rare and common variants in *KIF15* contribute to genetic risk of idiopathic pulmonary fibrosis. Am J Respir Crit Care Med.

[11] Zhang D, Newton CA, Wang B, Povysil G, Noth I, Martinez FJ (2022). Utility of whole genome sequencing in assessing risk and clinically relevant outcomes for pulmonary fibrosis. Eur Respir J.

[12] Partanen JJ, Häppölä P, Zhou W, Lehisto AA, Ainola M, Sutinen E (2022). Leveraging global multi-ancestry meta-analysis in the study of idiopathic pulmonary fibrosis genetics. Cell Genom..

[13] Locke AE, Steinberg KM, Chiang CWK, Service SK, Havulinna AS, Stell L (2019). Exome sequencing of Finnish isolates enhances rare-variant association power. Nature.

[14] Kurki MI, Karjalainen J, Palta P, Sipilä TP, Kristiansson K, Donner KM (2023). FinnGen provides genetic insights from a well-phenotyped isolated population. Nature.

[15] Kaunisto J, Kelloniemi K, Sutinen E, Hodgson U, Piilonen A, Kaarteenaho R (2015). Re-evaluation of diagnostic parameters is crucial for obtaining accurate data on idiopathic pulmonary fibrosis. BMC Pulm Med.

[16] Raghu G, Remy-Jardin M, Myers JL, Richeldi L, Ryerson CJ, Lederer DJ (2018). Diagnosis of idiopathic pulmonary fibrosis. An official ATS/ERS/JRS/ALAT clinical practice guideline. Am J Respir Crit Care Med.

[17] Allen RJ, Stockwell A, Oldham JM, Guillen-Guio B, Schwartz DA, Maher TM (2022). Genome-wide association study across five cohorts identifies five novel loci associated with idiopathic pulmonary fibrosis. Thorax.

[18] Ding L, Li B, Yu X, Li Z, Li X, Dang S (2020). KIF15 facilitates gastric cancer via enhancing proliferation, inhibiting apoptosis, and predict poor prognosis. Cancer Cell Int.

[19] Qiao Y, Chen J, Ma C, Liu Y, Li P, Wang Y (2018). Increased KIF15 expression predicts a poor prognosis in patients with lung adenocarcinoma. Cell Physiol Biochem.

[20] Bidkhori G, Narimani Z, Hosseini Ashtiani SH, Moeini A, Nowzari-Dalini A, Masoudi-Nejad A (2013). Reconstruction of an integrated genome-scale co-expression network reveals key modules involved in lung adenocarcinoma. PLoS ONE.

[21] Borie R, Kannengiesser C, Sicre de Fontbrune F, Gouya L, Nathan N, Crestani B (2017). Management of suspected monogenic lung fibrosis in a specialised centre. Eur Respir Rev.

[22] Terwiel M, Grutters JC, van Moorsel CHM (2022). Clustering of lung diseases in the family of interstitial lung disease patients. BMC Pulm Med.

[23] Molina-Molina M, Borie R (2018). Clinical implications of telomere dysfunction in lung fibrosis. Curr Opin Pulm Med.

[24] Snetselaar R, van Batenburg AA, van Oosterhout MFM, Kazemier KM, Roothaan SM, Peeters T (2017). Short telomere length in IPF lung associates with fibrotic lesions and predicts survival. PLoS ONE.

[25] Dressen A, Abbas AR, Cabanski C, Reeder J, Ramalingam TR, Neighbors M (2018). Analysis of protein-altering variants in telomerase genes and their association with *MUC5B* common variant status in patients with idiopathic pulmonary fibrosis: a candidate gene sequencing study. Lancet Respir Med.

[26] Stock CJ, Sato H, Fonseca C, Banya WA, Molyneaux PL, Adamali H (2013). Mucin 5B promoter polymorphism is associated with idiopathic pulmonary fibrosis but not with development of lung fibrosis in systemic sclerosis or sarcoidosis. Thorax.

[27] Fingerlin TE, Murphy E, Zhang W, Peljto AL, Brown KK, Steele MP (2013). Genome-wide association study identifies multiple susceptibility loci for pulmonary fibrosis. Nat Genet.

[28] Peljto AL, Zhang Y, Fingerlin TE, Ma SF, Garcia JG, Richards TJ (2013). Association between the *MUC5B* promoter polymorphism and survival in patients with idiopathic pulmonary fibrosis. JAMA.

[29] Wei R, Li C, Zhang M, Jones-Hall YL, Myers JL, Noth I (2014). Association between *MUC5B* and *tert* polymorphisms and different interstitial lung disease phenotypes. Transl Res.

