# Supplementary material for: KIF15 missense variant is associated with the early onset of idiopathic pulmonary fibrosis
Source: Respir Res. 2023 Sep 30;24:240. doi: 10.1186/s12931-023-02540-0 (PMC10543873; doi:10.1186/s12931-023-02540-0)
Supplement: Supplementary file 1 — Additional file 1. Table S1. Characteristics of the MUC5B positive patients. [file 12931_2023_2540_MOESM1_ESM.docx]

|  |  | **MUC5B (N=97)** | **Others (N=38)** | ***sig.*** |
| --- | --- | --- | --- | --- |
| **Background & comorbidities** | |  |  |  |
|  | **Female sex** | 28 (29%) | 15 (40%) | 0·304 |
|  | **Male sex** |  |  | 0·304 |
|  | **Family history of pulmonary fibrosis** | 4 (4%) | 4 (11%) | 0·221 |
|  | **Smoking history** | 62 (64%) | 24 (63%) | 1·000 |
|  | **Cancers** | 27 (28%) | 6 (16%) | 0·183 |
|  | **BMI, kg/m²** | 27·5 (25·1–30·0) | 28·9 (25·0–30·6) | 0·439 |
|  | **Comorbidities, N** | 4·0 (2·0–6·0) | 4·0 (2·0–6·0) | 0·910 |
| **Treatment & prognosis** | |  |  |  |
|  | **Antifibrotic treatment** | 57 (59%) | 22 (58%) | 1·000 |
|  | **Lung transplantation** | 9 (9%) | 6 (16%) | 0·361 |
|  | **Oxygen therapy** | 28 (29%) | 15 (40%) | 0·304 |
|  | **Acute exacerbations** | 25 (26%) | 9 (24%) | 1·000 |
|  | **Deceased** | 46 (47%) | 16 (42%) | 0·701 |
|  | **Age at diagnosis, years** | 71·0 (66·0–75·0) | 71·0 (62·5–78·0) | 0·922 |
|  | **Age at death, years** | 78·7 (73·9–82·5) | 79·1 (74·1–84·3) | 0·499 |
|  | **Age at transplant, years** | 63·5 (61·0–65·2) | 49·6 (44·1–57·1) | **0·026*** |
|  | **Age at death or transplant, years** | 76·6 (68·7–81·1) | 74·3 (60·8–83·0) | 0·644 |
| **Symptoms at diagnosis** | |  |  |  |
|  | **Dyspnoea** | 48 (50%) | 20 (53%) | 0·849 |
|  | **Cough** | 52 (54%) | 17 (45%) | 0·444 |
| **Laboratory findings at diagnosis** | |  |  |  |
|  | **Macrocytosis** | 22 (23%) | 8 (22%) | 1·000 |
|  | **Thrombocytopenia** | 13 (13%) | 5 (14%) | 1·000 |
| **Radiological findings at diagnosis** | |  |  |  |
|  | **Traction bronchiectasis** | 94 (97%) | 38 (100%) | 0·559 |
|  | **Honeycombing** | 81 (85%) | 27 (71%) | 0·150 |
|  | **Ground-glass opacity** | 18 (19%) | 7 (18%) | 1·000 |
|  | **Right ventricular strain** | 14 (14%) | 3 (8%) | 0·395 |
|  | **Emphysema** | 26 (29%) | 6 (16%) | 0·260 |
| **Pulmonary function at diagnosis** | |  |  |  |
|  | **FVC, L** | 3·04 (2·52–3·73) | 2·88 (2·32–3·59) | 0·348 |
|  | **FVC, % predicted** | 82·0 (68·0–92·5) | 81·0 (65·8–95·5) | 0·957 |
|  | **FEV1, L** | 2·49 (2·04–3·00) | 2·32 (1·91–3·00) | 0·405 |
|  | **FEV1, % predicted** | 80·0 (73·0–95·0) | 81·0 (71·3–90·3) | 0·606 |
|  | **DLCO, % predicted** | 59·0 (50·0–70·0) | 60·5 (54·5–69·0) | 0·311 |
| Data are n (%) or median (IQR), and p<0·05 is marked with *. BMI = body mass index. FVC = forced vital capacity. FEV1 = forced expiratory volume in 1 second. DLCO = diffusing capacity of the lung for carbon monoxide. | | | | |
